# Unmasking the mimic: lipoid pneumonia imitating primary lung cancer - a case report series of a diagnostic challenge

**DOI:** 10.3389/fonc.2025.1538418

**Published:** 2025-03-28

**Authors:** Shehab Mohamed, Luca Bertolaccini, Mariano Lombardi, Clementina Di Tonno, Angela Sabalic, Monica Casiraghi, Lorenzo Spaggiari

**Affiliations:** ^1^ Department of Thoracic Surgery, IEO, European Institute of Oncology Scientific Institute for Research, Hospitalization and Healthcare (IRCCS), Milan, Italy; ^2^ Division of Pathology, IEO, European Institute of Oncology Scientific Institute for Research, Hospitalization and Healthcare (IRCCS), Milan, Italy; ^3^ Department of Oncology and Hemato-Oncology, University of Milan, Milan, Italy

**Keywords:** lipoid pneumonia, lung cancer, differential diagnosis, EBUS, thoracic surgery

## Abstract

**Introduction:**

Lipoid pneumonia is a rare inflammatory disease characterized by an abnormal deposition of lipids in the alveoli. It may manifest as pulmonary consolidation, simulating primary lung cancer on radiological imaging and an increased uptake on fluorine-18-fluorodeoxy-D-glucose (FDG) positron-emission tomography (PET)/computed tomography (CT). The confirmed diagnosis can be achieved only by microscopic examination of cytological or histological samples.

**Methods:**

This paper describes eight cases at a single center from 2016 to 2024 of lipoid pneumonia mimicking primary lung cancer and its risk factors. Samples were collected thanks to multidisciplinary evaluations using CT and FDG-PET/CT. The histopathological diagnoses were obtained with endobronchial ultrasound (EBUS), fine needle aspiration biopsy (FNAB), or, as a last resort, surgical resection.

**Results:**

Our cohort of patients confirms that lung masses with fat density and irregular margins are lipoid pneumonia’s most common findings. However, it can also present as a solid mass with no fat density. This condition must always be appropriately evaluated through a multidisciplinary approach, especially when excluding a neoplastic origin.

**Conclusion:**

In this paper, we present the largest case series of lipoid pneumonia mimicking primary lung cancer from a single center reported in the literature so far. This case series highlighted the critical role of a multidisciplinary approach, including radiologists and pathologists, in differentiating lipoid pneumonia from malignancy to ensure optimal patient management.

## Introduction

Lipoid pneumonia is a rare inflammatory disease characterized by an abnormal deposition of lipids in the alveoli. It was first described in 1925; however, to date, we still lack accurate epidemiological data (1). This condition can be classified based on the source of the lipids as exogenous or endogenous (2). Endogenous lipoid pneumonia can be triggered by the release of fats and cholesterol in response to inflammatory events such as alveolar proteinosis and bronchial obstruction, including those caused by lung cancer, pulmonary fat embolism, and hereditary fat metabolism disorders (2). In contrast, several risk factors have been described for exogenous lipoid pneumonia, including the inhalation of oil-based products, such as nasal decongestants, laxatives, or lubricants, or gastroesophageal reflux disease. Recently, the use of e-cigarettes (3) (electronic nicotine delivery systems or ENDS) and vaping have been described as risk factors. Lipid inhalation from the liquids used in ENDS (e.g., glycerin and flavoring agents) has been linked to the onset of lipoid pneumonia (4). Furthermore, the use of ENDS has also been associated with the development of EVALI (e-cigarette, or vaping, product use-associated lung injury), an acute lung injury characterized by the presence of lipid-laden macrophages (5). However, some authors argue that lipoid pneumonia may not be a true form of EVALI due to differences in radiological imaging (fat attenuation areas), pathogenetic mechanisms, and clinical course (4, 5).

Lipoid pneumonia may manifest as a pulmonary consolidation, especially in the chronic condition, which can simulate primary lung cancer on radiological imaging.

Patients presenting with symptoms usually have fever, dyspnea, and cough, similar to infectious pneumonia. Most cases present as an asymptomatic or paucisymptomatic condition with an aspecific chronic cough; for this reason, the incidence and the prevalence are unknown. Rana et al. reported an incidence of 1%–2.5% based on 15 autopsy specimens from a single institution between 1992 and 2001 (6).

Exogenous lipoid pneumonia may present as an acute/subacute or chronic condition, which have mostly been by case reports reported in the literature (7, 8). Baron et al., in their retrospective study, found significant differences between acute and chronic lipoid pneumonia. Acute lipoid pneumonia usually has a short duration of symptoms and a fever requiring oxygen therapy and may be caused by an aspiration of a large quantity of exogenous substance. These patients usually have a similar course to classic pneumonia when treated, followed by radiological improvement. However, chronic lipoid pneumonia may be found incidentally with no symptoms, and even after avoiding the exogenous substance, no radiological improvement is usually observed (9).

Inhaled oil products usually induce acute intra-alveolar inflammation characterized by oil phagocytation by macrophages, which results in vacuolization. This condition is diagnosed by chromatography, chemical analysis, and electron microscopy (10).

The radiological presentation of this condition may mimic pneumonia or malignancy. Chest x-ray and computed tomography (CT) scans may show a peripheral mass that can have an increased uptake on fluorine-18-fluorodeoxy-D-glucose (FDG) positron-emission tomography (PET)/CT due to the inflammatory reaction. The confirmed diagnosis can be achieved only by microscopic examination of cytological or histological samples.

This study aimed to analyze a series of eight cases from 2016 to 2024, the largest from a single center reported in the literature thus far, where lipoid pneumonia presented as a condition mimicking primary lung cancer. All the patients described in this case series were carefully selected from our database thanks to a multidisciplinary team based on the suspicion of lung cancer at the time of presentation. Every patient presented different clinical and radiological presentations. Endobronchial ultrasound-transbronchial needle aspiration biopsy (EBUS-TBNA), fine needle aspiration biopsy (FNAB), or surgical resection achieved a cytological or histological diagnosis.

The study sought to provide insights into improving diagnostic accuracy and preventing unnecessary invasive procedures or treatments.

## Case descriptions

### Case 1

**Figure 1:**
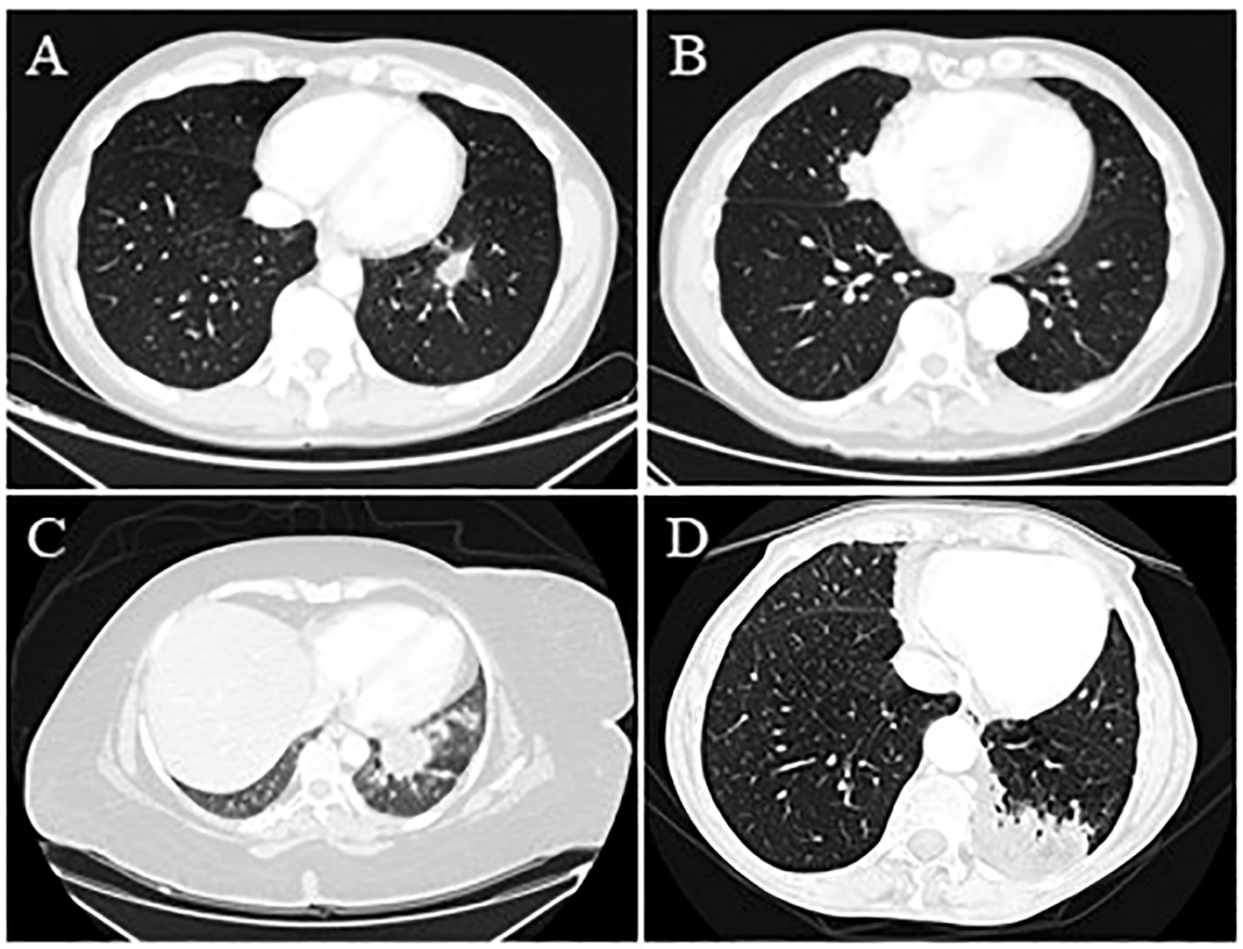
(Case 1: **A**) Chest CT scan shows a 26 mm x 16 mm lung mass in the left lower lobe with finely irregular specular margins; branches protrude into the neighboring parenchyma. The area also appears centrally connected with some vascular-bronchial afferents for the left lower lobe and is connected through branches with the hemidiaphragmatic and mediastinal pleura. In this context, the components are found to be hypodense. (Case 2**: B**) Lung consolidation area of the medial segment of the middle lobe with polylobulated profiles with a maximum size of 49 mm x 24 mm in the sagittal plane and contiguous to the right pericardial profile, suspected for primary lung cancer. (Case 3: **C**) A non-homogeneous contrast enhancement with low central attenuation characterized a pulmonary lesion measuring nearly 4 cm in the left lower lobe. (Case 4: **D**) A chest CT scan reported a 7 cm consolidative mass in the posterior left lower lobe and a station 5 aortopulmonary lymphadenomegaly with a central area of fat attenuation within the lesion.

A 53-year-old man, a former smoker with no significant past medical history, presented with a left lower lobe (LLL) nodule on a routine low-dose chest CT scan. A physical examination revealed no abnormalities. The CT showed a 26mmx16mm irregular nodule in the LLL (**Figure 1A**). On PET, the consolidation’s FDG uptake was slightly increased with a maximum standard uptake value (SUVmax) of 1.5. The patient underwent bronchoscopy and broncho-alveolar lavage (BAL), which were negative for malignancy. However, due to the radiological suspicion of malignancy and the limited diagnostic yield of BAL, a multidisciplinary board recommended a left basal segmentectomy. A histopathological examination revealed aggregation of vacuoles corresponding to exogenous lipoid pneumonia. In retrospect, a more detailed exposure history, obtained after surgery, reported that the patient had previously used a nasal decongestant (Rinostil oil-drops), which was likely the source of the exogenous lipid deposition. This highlights the importance of a thorough investigation into exposure history; a thorough preliminary investigation would undoubtedly have assisted the multidisciplinary team in interpreting the radiological consolidation, which was highly suspicious for malignancy. However, surgical treatment allowed for the definitive exclusion of malignancy and the management of lipoid pneumonia. The clinical course was uneventful. The patient only underwent a 1-month postoperative follow-up, which showed a radiological profile consistent with the surgical procedure.

### Case 2

An 81-year-old man, a former smoker, was referred to our department because of a middle lobe nodule (**Figure 1B**). He had a positive cardiological history of ischemic heart disease treated with percutaneous coronary intervention (PTCA) and two stents. The PET-FDG showed an increased uptake value in the nodule (SUVmax 10.96) and the bilateral hilar and mediastinal lymph nodes. The patient underwent EBUS, which was negative on the subcarinal and right paratracheal lymph nodes. Due to the strong suspicion of malignancy, the patient underwent middle lobe wedge resection through a thoracotomy approach because of dense adhesions. The histology report was consistent with fibrotic proliferation and chronic inflammatory infiltration in the interstitial and peribronchial spaces and rich in lympho-plasmacellular elements associated with macrophages and lipoid material. This histology was consistent with lipoid pneumonia. Six months after surgery, the patient was in good general condition, and the radiological assessment was consistent with the surgical resection.

### Case 3

A 40-year-old woman, a non-smoker with severe obesity, depression under treatment, and multiple episodes of aspiration pneumonia, was referred to our center for a 40mmx30mm left lower lesion on a CT scan with an increased contrast enhancement and suspicious for lung cancer (**Figure 1C**). The patient underwent complete staging with PET-FDG (positive, with no available SUVmax) and a TBNA, which was positive for lipoid pneumonia. Due to a new 19mm nodule appearing near the previous one, the patient underwent another TBNA with CT fluoroscopy guidance, which was again suggestive of lipoid pneumonia. Follow-up imaging at 1 year showed stable radiological findings, further supporting the benign nature of the previously described nodules.

### Case 4

A 70-year-old woman with a positive cardiological history of paroxysmal atrial fibrillation under treatment and hypertension underwent a CT scan after the onset of abdominal pain and diarrhea, which reported a 7 cm lung mass in the left lower lobe and a station 5 aortopulmonary lymphadenomegaly (**Figure 1D**). The PET-FDG was positive for the lesion (detailed SUVmax not available) and negative for the lymph nodes. The lung mass was assessed through transbronchial biopsies with fluoroscopy, which was negative, and FNAB, which was positive for lipoid pneumonia. The histological examination showed chronic granulomatous inflammation, which was not necrotizing, predominantly of the foreign body type and associated with giant cells. In the context of such cells and the surrounding parenchyma, optically empty microvesicles suggestive of lipoid origin were observed. This patient did not have any specific conditions predisposing her to lipid intake. Follow-up data were not available.

### Case 5

**Figure 2:**
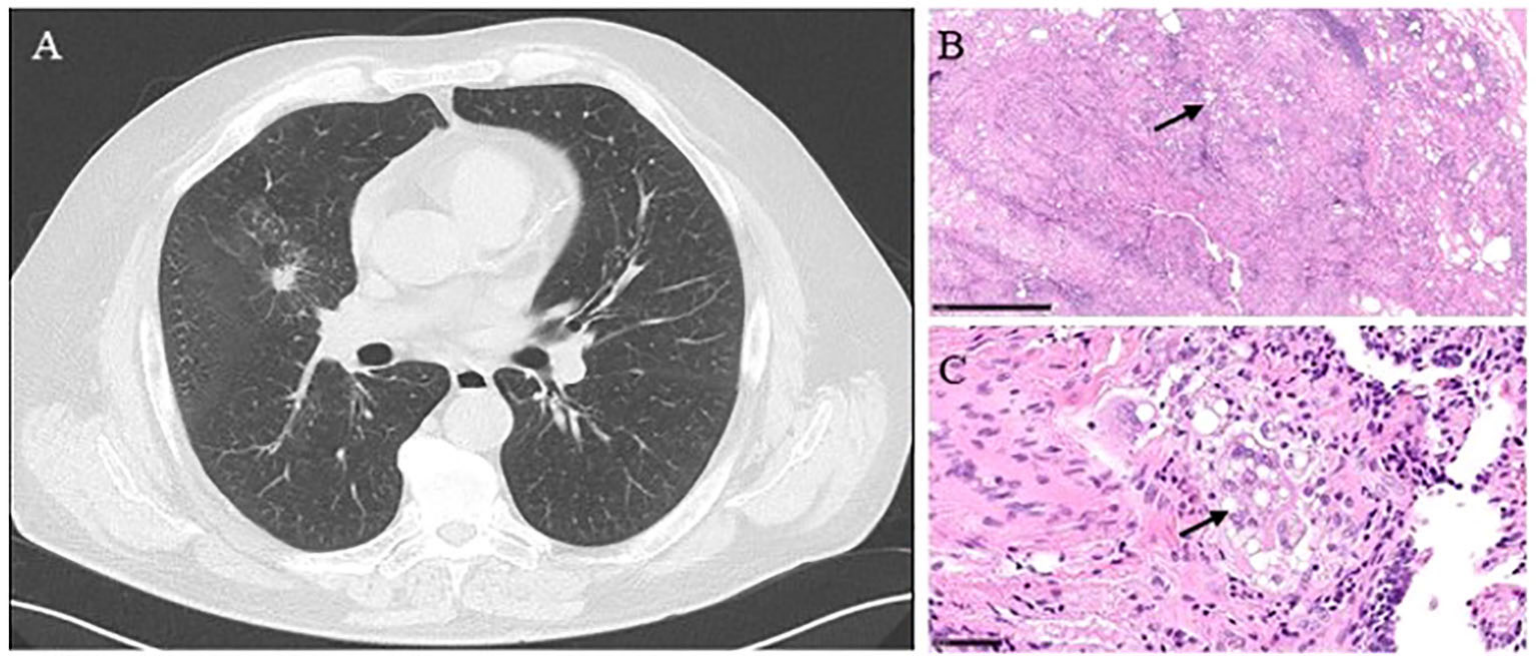
(Case 5) **(A)** A chest CT scan showing a 15 mm middle lobe lesion with low central attenuation and adjacent interstitial thickening. **(B)** The presence of lipoid material effaces the alveolar architecture. A combination of lipid droplets (arrow) and fibroinflammatory reaction leads to the low-power appearance of multiple and variably empty cystic spaces—hematoxylin and eosin stain; x2 power. **(C)** Lipoid pneumonia shows numerous and variably sized lipid droplets within macrophages (arrow), multinucleated giant cells, and the interstitium. The inflammatory infiltrate is also present—hematoxylin and eosin stain; x15 power.

A 65-year-old man, an active smoker in good general condition, presented at the outpatient clinic with a CT scan showing a 15mm middle lobe lesion (**Figure 2A**). An attempted FNAB and EBUS had already been performed; unfortunately, both were non-diagnostic. The radiological aspect was strongly suspicious for primary lung neoplasm; for this reason, he completed staging with PET-FDG, which reported an increased uptake (SUVmax 1.7). The patient underwent a diagnostic middle lobectomy and lymphadenectomy through a robotic approach. The histological results for the consolidation and the lymph nodes revealed fibrosis and chronic gigantocellular infiltration due to a foreign body consistent with lipoid pneumonia (**Figures 2B, C**). Based on these results, the patient was further questioned about anamnesis, and he reported nasal decongestant use, which was stopped after the surgery. The 12-month follow-up showed a radiological profile consistent with the surgical resection performed. No new suspicious consolidations were detected over time.

### Case 6

**Figure 3:**
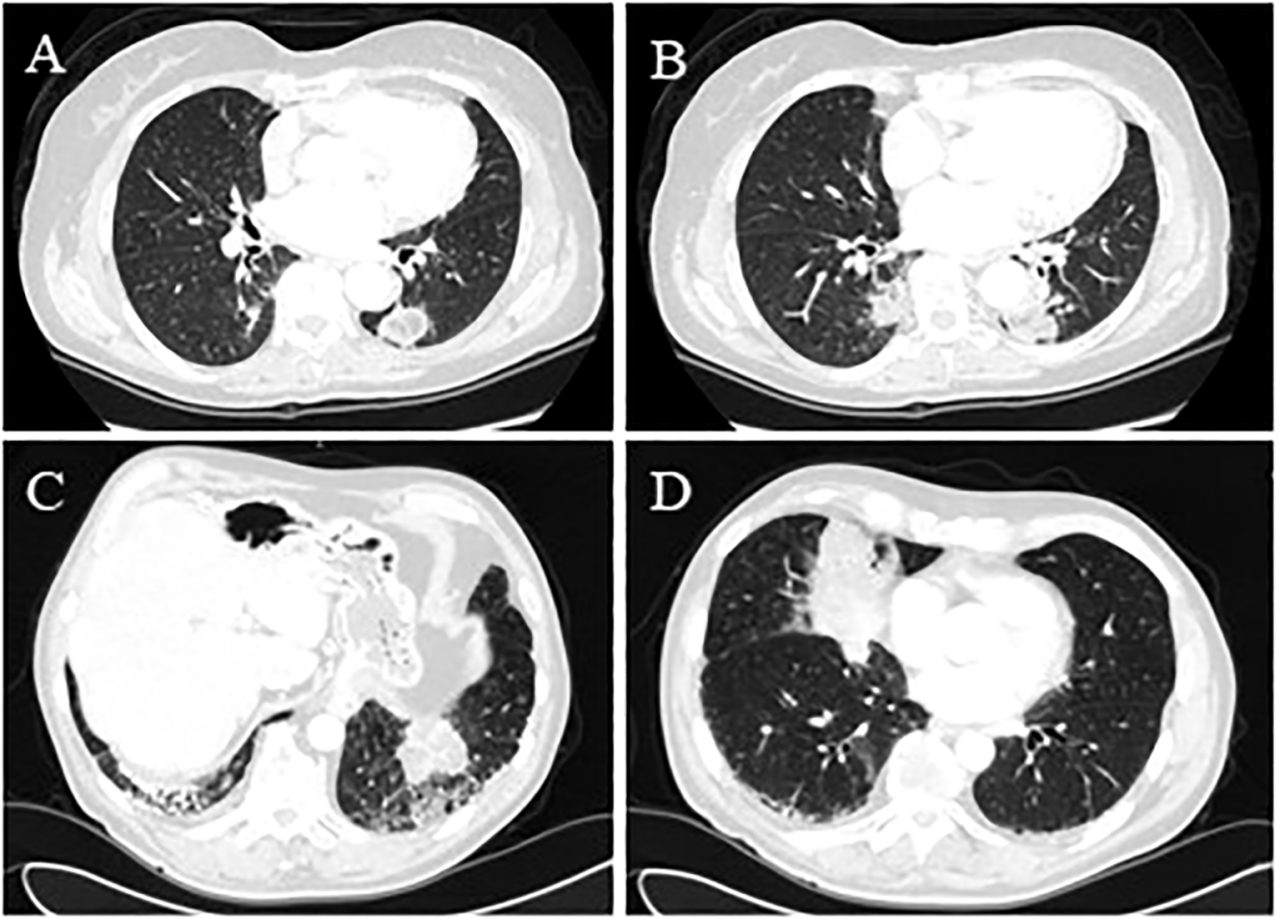
(Case 6: **A**) Bilateral pseudonodular lesions were consistent with lipoid pneumonia with low central attenuation. The lesion in the right lower lobe was 21 mm x 16 mm x 18 mm, which increased over time and was associated with ground-glass changes. (Case 6: **B**) The chest CT scan in the left lower lobe described a confluent aspect of the lung lesions in a 33 mm x 24 mm x 35 mm pseudonodular image with irregular margins. (Case 7: **C**) Bilateral lung lesions, which measured nearly 6 cm and were located in the postero-basal segment of the left lower lobe adjacent to bronchiectasis and reticulum-nodular thickening of the interstitium. (Case 7: **D**) The middle lobe lesion measured nearly 8 cm and was associated with ground-glass changes.

A 76-year-old woman, a former smoker (35 packs/year), was referred to our center for a long history of bilateral lung consolidation (**Figures 3A, B**). She had a past medical history positive for breast cancer 10 years earlier, treated with surgery and radiotherapy. Both the consolidations were radiologically suspicious for lipoid pneumonia, and the patient had also revealed usage of a nasal decongestant (hypertonic solution at 2.2% with hyaluronic acid). During the radiological follow-up, the left lung consolidation became more solid and positive for PET-FDG (detailed SUVmax not available). For this reason, the patient underwent BAL, which was negative, and a sequential FNAB for both the consolidation, which was positive for lipoid pneumonia. The use of nasal decongestants raised the suspicion of lipoid pneumonia. However, since lipoid pneumonia is a rare condition, the patient’s oncological history and the highly suspicious radiological imaging led the multidisciplinary team to a non-surgical biopsy, which allowed for the histological diagnosis. Follow-up data were not available.

### Case 7

This case was a 69-year-old man with a positive medical history of multiple sclerosis, large bowel cancer treated with hemicolectomy 24 years earlier, chronic obstructive pulmonary disease, and polyarthralgia presented with bilateral lung consolidation with no lymphadenopathy (**Figures 3C, D**). Given the previous oncological history and the radiological findings, a neoplastic process was initially suspected, so he underwent PET-FDG that showed an increased uptake (SUVmax not available), followed by EBUS. The cytopathological report revealed lipid-laden lung macrophages with an accumulation of intracytoplasmic membrane-bound vacuoles consisting of lipoid pneumonia. The absence of a history of lipid aspiration or exposure to exogenous lipids made the differential diagnosis between malignancy and inflammation more challenging, particularly given the overlap in radiological imaging and PET-FDG findings in both conditions. However, EBUS provided a definitive diagnosis, eliminating the need for more invasive surgical procedures. Follow-up data were not available.

### Case 8

**Figure 4:**
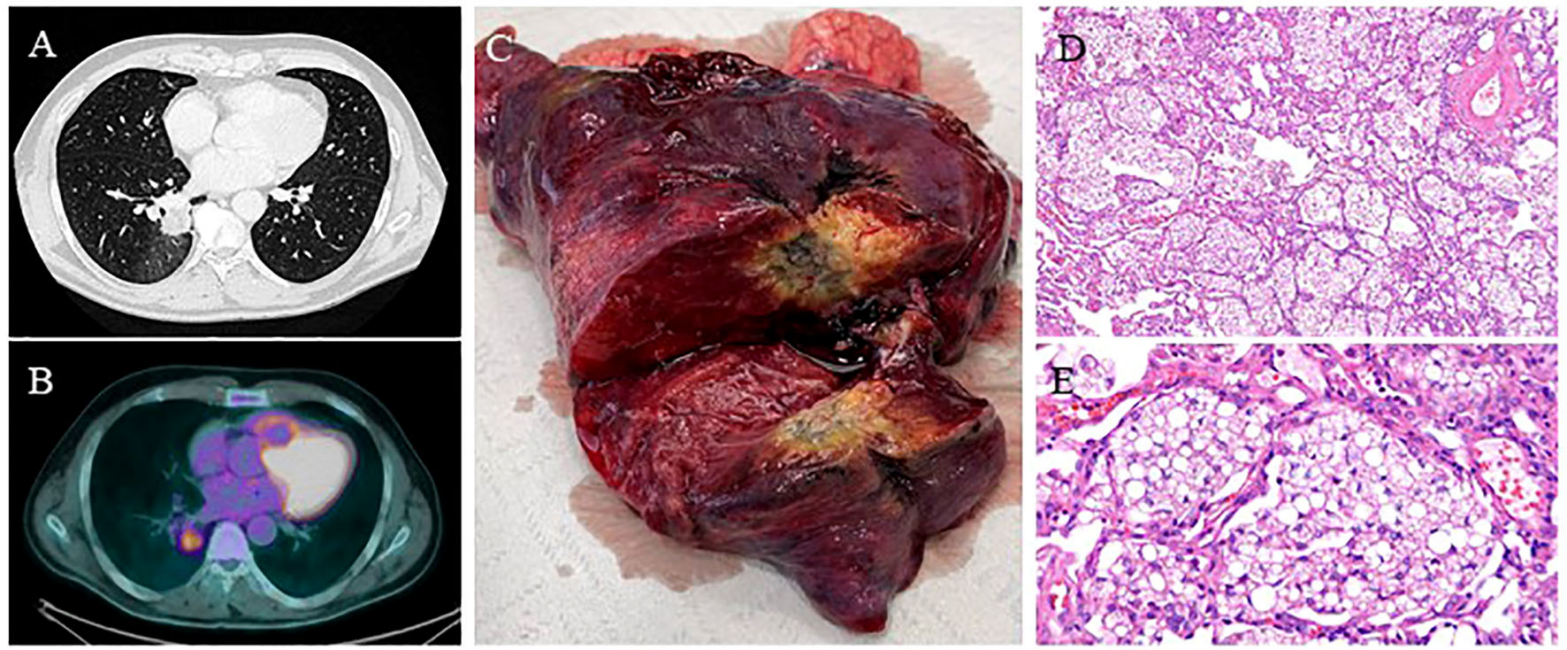
(Case 8**: A**) Chest CT scan showing a spiculated 31 mm right lower lobe lesion. **(B)** PET-FDG showing lung lesion uptake with a SUVmax of 4.8. **(C)** The right lower lobe specimen shows the known lung lesion. **(D-E)** Pathology reported an extensive chronic interstitial granulomatous inflammation with multinucleated foreign body giant cells, areas of stromal fibrosis, and alveolar structures containing abundant lipoid-like material and histiocytes. Moderate chronic inflammation is associated with follicular lymphoid aggregates. Sinus histiocytosis, anthracosis, and lipoid-like material in the hilar/peribronchial lymph nodes were examined—hematoxylin and eosin stain; x10 power and x40 power.

This case was a 53-year-old man, a former smoker (20 packs/year) with a positive medical history of sleep apnea and multiple related procedures, such as tonsillectomy, adenoidectomy, and rhinoplasty, chronic use of nasal decongestants (hypertonic solution at 2.2% with hyaluronic acid and nafazolina nitrato 1 mg). He presented to medical attention due to deep vein thrombosis. The patient was then referred to thoracic surgery. A CT scan of the chest showed a 31mm right lower lobe lesion, suspicious for primary lung cancer. The total body CT scan did not show any other relevant findings, and PET-FDG was positive at the lesion site with an SUVmax 4.8 (**Figures 4A, B**). The lung lesion was then assessed through transbronchial biopsies with fluoroscopy, which was negative for malignancy. Due to the high radiological suspicion of lung cancer and the very central position of the lesion, the patient underwent a robotic-assisted right lower lobectomy. The histological examination showed extensive chronic interstitial granulomatous inflammation with areas of stromal fibrosis and alveolar structures containing abundant lipoid-like material and histiocytes. Lipoid-like material was also found in lymph nodes (**Figures 4C–E**). After the intraoperative diagnosis, further interviews revealed a chronic intake of high-dose oil-based nasal lubricants. Each patient with a history of exposure to exogenous lipids was advised to stop any intake of the etiological factor. The 6-month follow-up showed a radiological profile consistent with the surgical procedure performed, with no evidence of new lesions.

## Discussion

Lipoid pneumonia is a rare condition characterized by an accumulation and deposition of exogenous or endogenous lipids that cause inflammation of the lung parenchyma. The prevalence is unknown because it is a chronic and asymptomatic condition. Few studies have been able to reach a diagnosis, with an incidence of 1%–2,5% postmortem, which reveals lipid material in the lung parenchyma (3, 6). This condition may also soon increase in incidence and prevalence due to the increased worldwide use of e-cigarettes, which is a documented risk factor for the inhalation of heated oil-based aerosols (11).

There is no pathognomonic radiographic finding of lipoid pneumonia. Although several CT and PET-FDG findings have been described in the literature, only very few case reports have shown lipoid pneumonia simulating lung cancer.

Indeed, low density in a lung mass on a CT scan can be represented by fat and, at the same time, necrotic tissue and mucous retention. This non-specific finding can be seen in other benign conditions such as lipoma and hamartoma. However, increased FDG uptake in lipoid pneumonia has been described, thus simulating a tumor. In many cases, cytological or histological samples had to be evaluated to achieve a final diagnosis (12–14).

In general, chest CT may show areas of fat attenuation with values lower than −30 HU (Hounsfield) associated with opacities and nodules. Opacities are typically ground-glass or mass-like consolidations and can have different distributions, ranging from bilateral to lobar, predominantly in the lower lobes, or segmental. Other possible radiological manifestations could be pneumatoceles, pneumomediastinum, pneumothorax, and pleural effusions (15, 16).

One diagnostic feature, especially for exogenous lipoid pneumonia, is the presence of fat within the mass, which is speculated to be due to chronic inflammation and fibrosis. Sometimes, the fat-containing mass can be misdiagnosed as hamartomas or liposarcomas.

Many reasons can cause the mass to be misdiagnosed as lung cancer, such as the absence of fat and the increased uptake of the FDG on PET due to the inflammation within the mass. These manifestations remain stable even if the patient stops consuming mineral or vegetable oils.

The findings of this case series underscore the critical importance of maintaining a high index of suspicion, particularly in patients with atypical presentations or risk factors for lipoid pneumonia, such as exposure to exogenous lipids or underlying chronic pulmonary conditions. Failure to accurately diagnose this entity can lead to inappropriate interventions, including unwarranted invasive procedures or surgical resection, which could have been avoided with a more precise diagnosis.

In this study, of eight patients, four (50%) had a history of nasal decongestant or oil-based nasal lubricant intake, and one had several episodes of aspiration pneumonia, which are well-known risk factors for exogenous lipoid pneumonia.

In prolonged nasal spray or decongestant intake, the patient may develop chronic lipoid pneumonia, which can be asymptomatic and only detected as an incidental finding on chest x-rays or CT scans for other reasons, as in cases 7 and 8. In the acute form, lipoid pneumonia may be diagnosed due to the onset of fever, cough, and dyspnea due to a large intake of oil products.

Similar to previous studies (2, 17–28), our cohort of patients confirms that lung masses with fat density and irregular margins are the most common findings of lipoid pneumonia. However, it can also present as a solid mass with no fat density.

This condition must always be appropriately evaluated through a multidisciplinary approach (29), especially to exclude neoplastic origin. Radiologists have a key role in the initial diagnosis based on the CT scan. Samhouri et al. emphasize the importance of fat attenuation on CT, observed in 23% of patients with histologically confirmed lipoid pneumonia and in 41% of their entire cohort (30). If the CT scan or PET-FDG cannot diagnose the lung mass and distinguish pneumonia from malignancy, it has to be evaluated by a tissue sample, especially if there is strong suspicion, which can be collected using EBUS-TBNA, FNAB, or, in a last resort, surgical resection. In our case series, histopathological evaluations revealing lipid-laden macrophages provided the definitive diagnoses, avoiding the risks and complications of an unnecessary thoracotomy or lobectomy. This case series emphasizes the need to integrate clinical history, imaging findings, and histopathological confirmation when establishing an accurate diagnosis (**Table 1**). Clinicians must remain vigilant about the possibility of lipoid pneumonia, especially in cases with a history of chronic aspiration, oil-based medication use, or occupational exposure. Early recognition and diagnosis are crucial, as management typically involves supportive care and addressing the underlying cause rather than aggressive oncological treatments. However, as demonstrated by Samhouri et al. in a cohort of 34 patients, in 89% of cases, the causative substance was identified only after the diagnosis (30).

**Table 1:** Comparison of key features between lipoid pneumonia and primary lung cancer.

Characteristic	Lipoid pneumonia	Primary lung cancer
Etiology	Exposure to exogenous lipids (e.g., oil-based substances), aspiration	Tobacco use, genetic mutations, environmental carcinogens
Clinical presentation	Cough, dyspnea, chest pain (may be subacute or chronic)	Cough, weight loss, hemoptysis, fatigue
Radiological features	Consolidation, nodular opacities, ground-glass changes	Irregular mass, cavitation, lymphadenopathy
Histopathology	Lipid-laden macrophages, granulomatous inflammation	Malignant cells, desmoplastic stroma
Diagnostic approach	CT, biopsy showing lipid-laden macrophages	CT, PET scan, biopsy confirming malignancy
Management	Supportive care, addressing underlying cause	Surgery, chemotherapy, radiotherapy, targeted therapies
Prognosis	Good with appropriate management	Variable depending on stage and treatment modality

Patients undergoing diagnosis with a history of oil-based products inhalation or ingestion must stop any intake of the etiological factor, and in specific cases, symptomatic treatment with oxygen, broad-spectrum antibiotics, and corticosteroid therapy may be necessary. These patients may also undergo a radiological follow-up with a CT scan to evaluate the evolution over time to ensure that a lung cancer diagnosis is not missed, especially in high-risk patients.

The study has several limitations that warrant consideration. First, the condition’s rarity limits our observations’ generalizability to broader populations. Second, there may be variability in the imaging techniques and histopathological evaluations across the cases, potentially influencing the consistency of diagnostic interpretations. Third, the study does not address long-term outcomes. These limitations highlight the need for further research, preferably through multicenter studies with standardized protocols.

## Conclusion

Lipoid pneumonia is a rare condition that can be classified as exogenous or endogenous based on the source of the lipids. The symptoms may vary from acute forms, in which the clinician can identify the cause more quickly, to subacute or chronic forms, in which the diagnosis is much more complex due to a lack of personal history, nuanced symptoms, and non-pathognomonic radiological findings. In the latter case, diagnosis may be delayed, and a multidisciplinary evaluation is also needed to avoid unnecessary examinations. If lipoid pneumonia is suspected, a more careful revision of the anamnesis is advised to investigate risk factors that may have initially been underestimated.

The case series highlighted the critical role of a multidisciplinary approach, including radiologists and pathologists, in differentiating lipoid pneumonia from malignancy to ensure optimal patient management.

## Data Availability

The raw data supporting the conclusions of this article will be made available by the authors, without undue reservation.
